# Mitochondrial DNA Lineages of Eurasian Otters (*Lutra lutra*) in Central Anatolia: A Phylogenetic Bridge Between Asia and Europe?

**DOI:** 10.3390/ani16213371

**Published:** 2026-10-26

**Authors:** Emrah Aslan, Yasin Demirbaş, Ayça Özkan Koca, Kubilay Toyran, Hakan Soysal, Franz Suchentrunk

**Affiliations:** 1 Department of Biology, Faculty of Engineering and Natural Sciences, Kırıkkale University, Kırıkkale 71450, Türkiye; emrahaslan@kku.edu.tr (E.A.); yasdemirbas@kku.edu.tr (Y.D.); hsoysal50@msn.com (H.S.); 2 Department of Gastronomy and Culinary Arts, Faculty of Fine Arts, Maltepe University, İstanbul 34857, Türkiye; aycakoca@maltepe.edu.tr; 3 Department of Medical Services and Techniques, Şabanözü Vocational School, Çankırı Karatekin University, Çankırı 18650, Türkiye; kubilaytoyran@karatekin.edu.tr; 4 Research Institute of Wildlife Ecology, University of Veterinary Medicine Vienna, 1160 Vienna, Austria; 5 Wildlife Research—Vienna & Bredasdorp, Bredasdorp 7280, South Africa

**Keywords:** Eurasian otter, *Lutra lutra*, MtDNA sequences, phylogeny, phylogeography, Kızılırmak River, Anatolia

## Abstract

The Eurasian otter (*Lutra lutra*), a semi-aquatic carnivore listed as “Near Threatened” by the IUCN (International Union for Conservation of Nature), is widespread across Eurasia but remains poorly studied genetically in various regions. We analyzed the Central Anatolian population along the Kızılırmak River using mitochondrial (mt) DNA from non-invasively collected samples to examine whether Anatolian otters connect phylogenetically typical European and Caucasian lineages and whether Anatolia could have harbored a late-glacial source population for otter migration to Europe via western (Balkan) and/or eastern (east of the Black Sea) routes. Thirty spraints were collected from five localities, including four previously known sites and one newly identified in the Sulakyurt district, northeast of Kırıkkale province. We amplified a short (256 bp) mtD-loop fragment and a longer (820 bp) fragment spanning the *Cytb*, tRNA-Thr, tRNA-Pro, and D-loop from 14 samples and included all corresponding *Lutra lutra* mtDNA sequences available in GenBank in our analyses. Six short-fragment samples from Kapulukaya, Kesikköprü, and Kalecik carried the widespread northern Eurasian haplotype Lut1, while eight samples from Kalecik, Kesikköprü, Hirfanlı, and Hamzalı revealed a novel haplotype (H256-TR). The long fragments represented two additional haplotypes (H820-37, H820-38), distributed across the same localities. Median-joining network analysis of the short fragment produced a star-like topology with no clear phylogeographic structuring, whereas the longer fragment resolved two weakly separated phylogenetic groups. Central Anatolian haplotypes were positioned between Far Eastern and northern Eurasian lineages, with H820-38 phylogenetically connecting Caucasian and European haplotypes. This finding is consistent with the hypothesis of possible western and eastern postglacial migration routes from Anatolia to Europe, although broader spatial sampling is needed for confirmation.

## Simple Summary

Given that only three Eurasian otter (*Lutra lutra*) samples from Türkiye have previously been sequenced, our present study provides some novel phylogeographic insights into the genetic diversity of Central Anatolian otters by analyzing mitochondrial (mt)DNA from non-invasive spraint samples along the Kızılırmak River. By sequencing short (256 bp) and long (820 bp) mtDNA fragments, novel haplotypes were identified alongside the widespread northern Eurasian “Lut1” lineage, indicating undocumented diversity and Anatolia’s potential role as a genetic reservoir. Median-joining network analysis demonstrated that this Central Anatolian population includes a haplotype that holds an evolutionary position between Far Eastern and European haplogroups. This supports the hypothesis that Anatolia may have acted as a late Pleistocene refugium during the Last Glacial Maximum and as a source population for postglacial northward gene flow. Standard (tree-building) phylogenetic and short-sequence network analyses, however, did not result in meaningful phylogeographic information. The close genetic affinities of Anatolian, Caucasian, and European haplotypes are concordant with the earlier view that *Lutra lutra meridionalis* from the Caucasus and the Middle East may be grouped into *L. l. lutra*. Our findings offer a starting point for understanding the hypothesized phylogeographic role of Anatolia in postglacial otter migration pathways to Europe and in future conservation genetic studies of this aquatic flagship species in the Middle East.

## 1. Introduction

The Eurasian otter (*Lutra lutra*) has the widest geographic distribution among all semi-aquatic mustelids and inhabits freshwater and coastal environments of remarkable diversity, including habitats in human-dominated landscapes that have undergone massive anthropogenic modifications—e.g., [1,2]. Because of its public appeal for raising conservation awareness and its sensitivity to pollution and habitat degradation, it serves as a “flagship species”, and because of its role in comprehensive regional habitat-specific and larger ecosystem-wide conservation of aquatic ecosystems, it serves as an “umbrella species” (see, e.g., [3], for a theoretical framework for “umbrella species” and [4,5], among others, in which the authors considered Eurasian otters “flagship species”) for conservation of aquatic ecosystems. Connective freshwater and riparian habitats as well as marine coastal habitats enable dispersal, recolonization, genetic exchange and access to diverse resources (e.g., [6] and the literature cited therein), whereas fragmented habitats caused by dams, urban development and the like may reduce their genetic diversity and long-term population persistence or viability. For instance, the authors of [7] concluded that habitat suitability and connectivity are essential for successful recolonization of otters in Alpine regions. Successful long-term colonization of regions and the associated historic background are best understood by analyzing the specific genetic make-up of regional populations. However, throughout their wide distribution across the Palearctic, the Middle East, South and Southeast Asia, and the northwest African Maghreb, many otter occurrences remain poorly characterized genetically, particularly in regions where populations may be threatened by recent habitat deterioration or fragmentation and increasing pollution, such as in Anatolia.

Otters have been recorded at generally low densities across nearly all of Türkiye, inhabiting streams, creeks, rivers, lakes, and coastal habitats [8,9,10,11,12]. The first documented record in Türkiye was provided in 1877 from Mersin–Tarsus [13]; another, much later report was from Muğla–Fethiye, and it described the fur characteristics of otter [14]. Otters were also observed in the Aras and Kura Rivers in the eastern Anatolian province of Kars [15], and further records have been published from other parts of Anatolia [16,17,18]. Most studies consist only of distributional records and observational bioecological research [9,10,11,19,20,21,22,23,24,25,26,27,28,29]. Immediate studies on the conservation of otters in Türkiye are very scarce, and genetic data are almost entirely absent, apart from mitochondrial (mt) haplotypes of three individuals (see below).

The authors of [30] investigated the effect of habitat quality on otter abundance in a small study area along the Kızılırmak River in Central Anatolia. Further, habitat quality indices were correlated with otter habitat use, and it was suggested that such indices could be used in establishing management strategies for otters. Further, the authors of [25] conducted direct observations in seven different regions to determine the activity areas of otters between February 2005 and August 2009 along approximately 130 km of the Kızılırmak River between the Hirfanlı Dam and the border of Kalecik district in Central Anatolia. Notably, dams, habitat loss, traffic accidents, fishing activities, recreational areas, and soil and water pollution were considered the most significant factors threatening the otter population.

Taxonomic studies on otters in Türkiye are solely based on morphological characteristics [21,25,31]: skull and fur characteristics of five females and four males obtained from different localities in the Elazığ and Malatya provinces in southeastern Anatolia revealed statistically significant sex-specific differences in skull characteristics, specifically in exorbital width and upper molar row length [31]. Anatolian otters are commonly considered to belong to *L. l. lutra*, the nominal subspecies with a Europe-wide distribution. Caucasian otters, i.e., from east and northeast of Anatolia, that are considered a separate subspecies—*L. l. meridionalis—*are not particularly different from the nominate species according to skull characteristics [32]; however, insufficient genetic information on otters in Türkiye is available for firm intraspecific/taxonomic conclusions.

In fact, information on the genetic diversity and intraspecific phylogeny of otters in Türkiye is limited to two haplotypes identified from the complete mitochondrial (mt) cytochrome b gene region (1140 bp) of three individuals sampled from the Kars, Sinop, and Istanbul regions [33]. Their evolutionary relationship to seven otter haplotypes downloaded from GenBank indicated two main haplogroups: one for the haplotypes from Türkiye, the Iberian Peninsula, Norway, and Poland and a second consisting of Korean haplotypes only. The presence of two different haplotypes of the western Palearctic haplogroup in the three Turkish otter samples suggested, however, the existence of at least two distinct lineages in Türkiye [33]. The positions of the Turkish haplotypes in the network led to the hypothesis that Anatolia may have served as a late Pleistocene (late glacial) refugium for otters. From there, two migration routes may have contributed to the postglacial colonization of Europe: one via the Balkans towards central Europe, and another from Anatolia to Eastern Europe through the (western) Caucasus and northward east of the eastern Black Sea coast [33]. Phylogeographically, this hypothesis is plausible given that Anatolia represents a key biogeographic crossroads between Europe, the Caucasus, and southwestern Asia, accommodating Euro-Siberian, Sindo-Turanian, and Arabic-African elements in its mammalian fauna—see, e.g., [34,35,36,37,38] for ranges of mammalian species.

In this study, we present the first non-invasive genetic assessment of otters along the Kızılırmak River in Central Anatolia. We sequenced two mtDNA fragments and compared them with all publicly available Eurasian otter reference sequences to evaluate the phylogenetic position of otters in this region.

We hypothesized that otters from the Kızılırmak Basin belong to the western Eurasian/Northern Palearctic mt lineage system [39] and may show affinities to both European and Caucasian haplotypes. Such a pattern would be consistent with the concept that Anatolia has acted as a phylogeographic linkage zone between Europe and the Caucasus and may have hosted a late-glacial refugial population contributing to postglacial gene flow to Europe.

We further investigated whether any newly detected Anatolian haplotypes align with the range expansion of mtDNA lineage 3, as proposed by the inclusion of otters from Iraq [39]. Moreover, our data may also provide information to evaluate the subspecific status of the mainly Caucasian *L. l. meridionalis*, which has been questioned recently [32].

Given the shallow level of differentiation of mtDNA hitherto observed in Eurasian otters, specifically for short-sequence alignments—e.g., [4,33,39]—we expected little or no conclusive phylogeographical resolution with short (256 bp) mt control-region sequences but some resolution with longer (820 bp) sequences of mtDNA, covering parts of the control region (CR) and the *Cytb* gene and including two genes coding for tRNA. Moreover, we expected more phylogeographical resolution by median-joining (MJ) networks that allow for alternative evolutionary pathways, rather than by tree-building approaches that force all evolutionary haplotype differences into hierarchically bifurcating terminal lineages where there may be no such hierarchies [40].

## 2. Materials and Methods

### 2.1. Study Area

**Figure 1:**
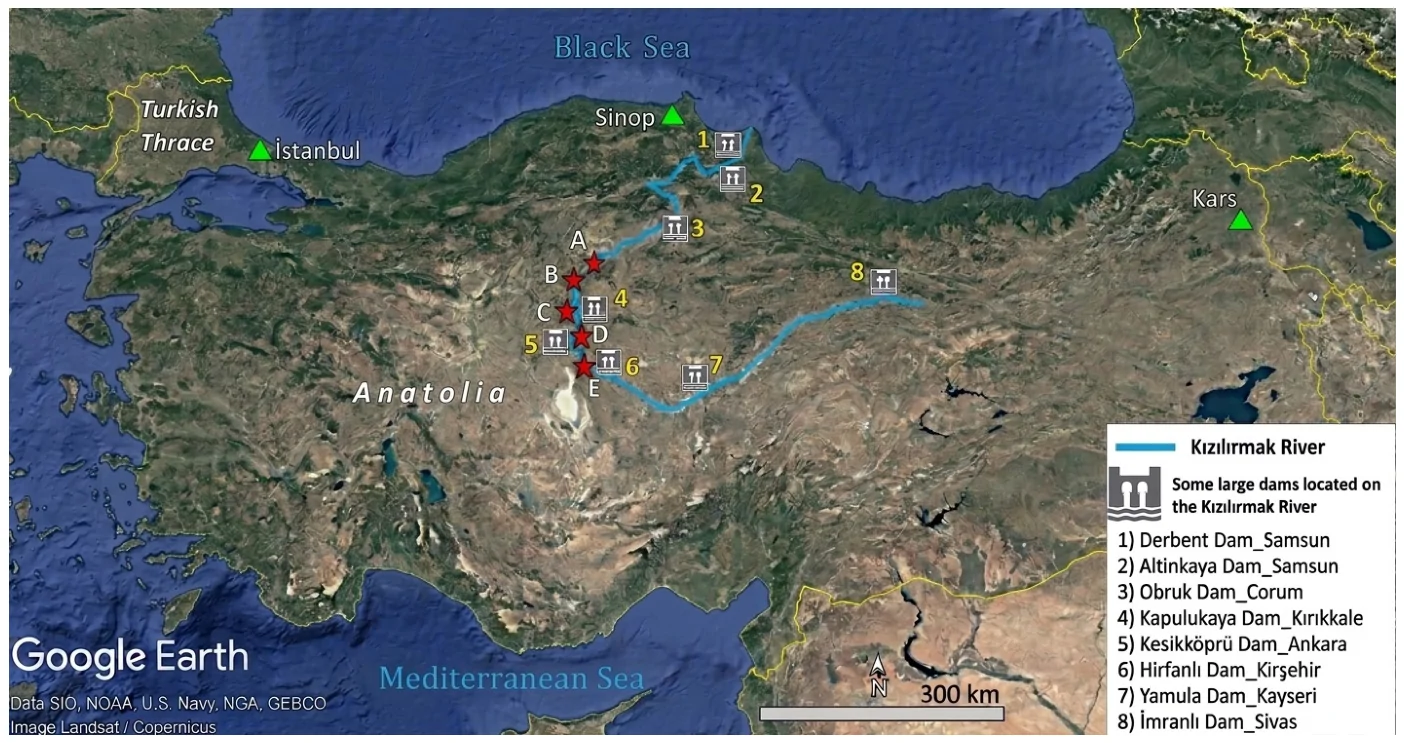
Locations of samples from Türkiye where mtDNA sequences of the Eurasian otter, *Lutra lutra*, were obtained. Red stars indicate the locations of spraint samples recorded from the Kızılırmak River in this study [A—Kırıkkale/Sulakyurt-Hamzalı Village; B—Ankara/Kalecik (Güriş Hydroelectric Power Plant); C—Kırıkkale/Hacılar (Kapulukaya Dam); D—Ankara/Bala (Kesikköprü Dam); E—Kırşehir/Kaman (Hirfanlı Dam]. Green triangles represent otter locations obtained by [33].

The Kızılırmak River is the longest river in Türkiye. Its length is approximately 1355 km, and it originates in the Kızıldağ region of Sivas province. It flows through the provinces of Sivas, Kayseri, Kırşehir, Kırıkkale, Ankara (short sections), Çankırı, Çorum, and Samsun before emptying into the Black Sea. There are eight major dams on the Kızılırmak River from source to mouth: İmranlı, Yamula, Hirfanlı, Kesikköprü, Kapulukaya, Obruk, Altınkaya, and Derbent. The study area covers five localities in the provinces of Ankara (2), Kırıkkale (2), and Kırşehir (1), through which the Kızılırmak flows. The length of the study area is approximately 160 km from Sulakyurt (A) to Hirfanlı Dam (E) (Figure 1). Perennial trees such as willows (*Salix* sp.) and poplars (*Populus* sp.), as well as blackberry bushes (*Rubus* sp.), dominate the riverbanks. However, reed, *Phragmites australis*, constitutes the general vegetation of the area.

### 2.2. Sampling

Fresh-looking otter spraints were collected along the Kızılırmak River on the 4th of September 2025 by two research groups in a single day. Sampling stations consisted of five river sections in Ankara, Kırıkkale, and Kırşehir provinces (study area length approximately 160 km), four of which were selected based on the results of previous studies [10]. Otter spraints were recorded for the first time at the Sulakyurt station in Kırıkkale province in this study (Table 1, Figure 1). By surveying approximately 1.5 km stretches at each site, the following numbers of fresh spraints were obtained: 2 in Sulakyurt, 8 in Kalecik, 6 at Kapulukaya Dam, 3 at Kesikköprü Dam, and 11 at Hirfanlı Dam.

Fresh spraints were collected on the same day from different geographically proximate localities to sample Eurasian otter individuals belonging to different populations. However, since multiple otter spraints were collected from each locality, individual identification could not be performed.

Fieldwork was conducted according to [41]. Riversides/banks were surveyed on a dry day in the early morning to prevent DNA degradation during the warmer hours of the day. The freshness of spraint samples was assessed visually, based on color and moisture. Fresh-looking spraints were immediately preserved in 95% ethanol and stored at −20 °C until analysis. As a result, we collected 30 otter spraints from four locations where records of their presence in the Kızılırmak River have existed since 2010 and from one newly identified location in Sulakyurt district, northeast of Kırıkkale province, on the Kızılırmak. All procedures contributing to this study were carried out within the scope of research permits obtained from the Turkish Ministry of Agriculture and Forestry, General Directorate of Nature Conservation and National Parks (permit no: E-72784983-288.04-20532558).

### 2.3. Genetic Analysis

DNA was extracted using the GeneMATRIX Stool DNA Purification Kit (EURx Ltd., Gdansk, Poland) after residual ethanol was removed under vacuum. The quality of the DNA samples was evaluated using 1.2% agarose gel electrophoresis.

For mtDNA analysis, we amplified and sequenced two fragments. The first fragment (256 bp) spanned the 5′ end of the control region (CR) and was used to compare our sequences with known *Lutra lutra* haplotypes available in the NCBI GenBank database (see GenBank accession numbers in Table S1). The second fragment (820 bp) included the 3′ end of the cytochrome b (*Cytb*) gene, tRNA-Thr, tRNA-Pro, and the 5′ end of the CR. This fragment was also used for comparison with known *L. lutra* haplotypes in the NCBI GenBank database (see GenBank accession numbers in Table S2). The two fragments were amplified using the primer pairs LlucybL996/H16498 [42] and Lut4F/Lut4R [43], respectively.

A 25 µL PCR mixture was prepared by combining 16 µL of distilled water, 1.25 µL each of the forward and reverse primers, 4.5 µL of PCR buffer [Solis Biodyne HOT FIREPol^®^ Blend Master Mix Ready to Load with 15 mM MgCl_2_, (Tartu, Estonia)] and 2 µL of DNA. The PCR cycling schedule for the LlucybL996/H16498 primer pair was 2 min at 94 °C; 40 cycles of three steps: 30 s at 94 °C, 30 s at 55 °C, 30 s at 72 °C; and 10 min at 72 °C [41]. The PCR cycling schedule for the Lut4F/Lut4R pair was 2 min at 94 °C; 40 cycles of three steps: 40 s at 94 °C, 40 s at 57 °C, 40 s at 72 °C; and 10 min at 72 °C [44]. Amplification was performed using a Kyratec Thermal Cycler (Kyratec Life Science, Seoul, Republic of Korea). The PCR products were visualized using electrophoresis in a 1.2% agarose gel and purified using ExoSAP (Fermentas, Waltham, MA, USA) following the manufacturer’s recommendations. Sequencing was conducted on an ABI 3100 Genetic Analyzer (Applied Biosystems, Waltham, MA, USA). Using the NCBI BLAST algorithm, all sequences obtained with the two different primer sets were confirmed to belong to *Lutra lutra*. The chromatograms were inspected using FinchTV v.1.4.0 to assess sequence quality and verify base calls. The sequences generated in this study were aligned using ClustalW implemented in BioEdit v.7.0.5.3 [45], with the default alignment parameters. The resulting alignments were manually inspected, and final adjustments were made by visual examination, particularly for the mitochondrial CR. Gaps/indels were retained where necessary to maintain positional homology. The final alignment lengths were 256 bp for the CR fragment and 820 bp for the fragment spanning the 3′ end of *Cytb*, tRNA-Thr, tRNA-Pro, and the 5′ end of the CR. The sequences generated in this study representing the three novel haplotypes have been deposited in GenBank under accession numbers QB048587 (CR fragment) and QB048588 and QB048589 (*Cytb*–tRNA-Thr–tRNA-Pro–CR fragment).

Median-joining (MJ) networks were constructed using NETWORK v10.2.0.0 [46]. Network calculations were performed with the default value for the epsilon parameter (ε = 0). Bayesian analysis was conducted in MrBayes 3.1.2 [47] under the K81uf+I+G nucleotide substitution model selected using Modeltest 3.7 [48] based on the Akaike Information Criterion (AIC). The Bayesian analysis included the *Lutra lutra* haplotypes derived from the 820 bp mitochondrial sequences, with *Lutra sumatrana* (GenBank accession number: NC_035810.1) used as the outgroup. Two independent runs, each consisting of four chains, were performed for 3 million generations, with sampling every 1000 generations. The first 25% of the sampled trees were discarded as burn-in. Convergence was assessed using the average standard deviation of split frequencies between the independent runs.

## 3. Results

The first dataset, based on the 256 bp long sequences of the mt CR, comprised 682 sequences, including 14 newly generated Turkish sequences and 668 reference sequences retrieved from GenBank, and yielded 30 unique haplotypes. The GenBank reference dataset included samples from Europe, Russia, Uzbekistan, China, Japan, and Korea. Among the Turkish sequences, six represented the widespread Eurasian haplotype Lut1, while eight formed the novel haplotype H256-TR (GenBank accession number QB048587). The second dataset, based on the 820 bp mitochondrial fragments, comprised 138 sequences, including 14 newly generated Turkish sequences and 124 reference sequences retrieved from GenBank, and yielded 39 unique haplotypes. The GenBank reference dataset included samples from Russia, the United Kingdom, China, and Korea. The newly generated Turkish sequences formed two novel haplotypes, H820-H37 (n = 8; GenBank accession number QB048588) and H820-H38 (n = 6; GenBank accession number QB048589) (Table 2). Detailed information on the haplotype assignments, geographic information, and reference sources of the sequences included in the two datasets are provided in Supplementary Materials (Tables S1 and S2).

Six short-fragment samples from Kapulukaya, Kesikköprü, and Kalecik carried the widespread northern Eurasian haplotype Lut1, while eight samples from Kalecik, Kesikköprü, Hirfanlı, and Hamzalı revealed the novel haplotype H256-TR. The haplotypes H820-H37 and H820-H38 (long fragments) were distributed across the same localities (Table 2).

**Figure 2:**
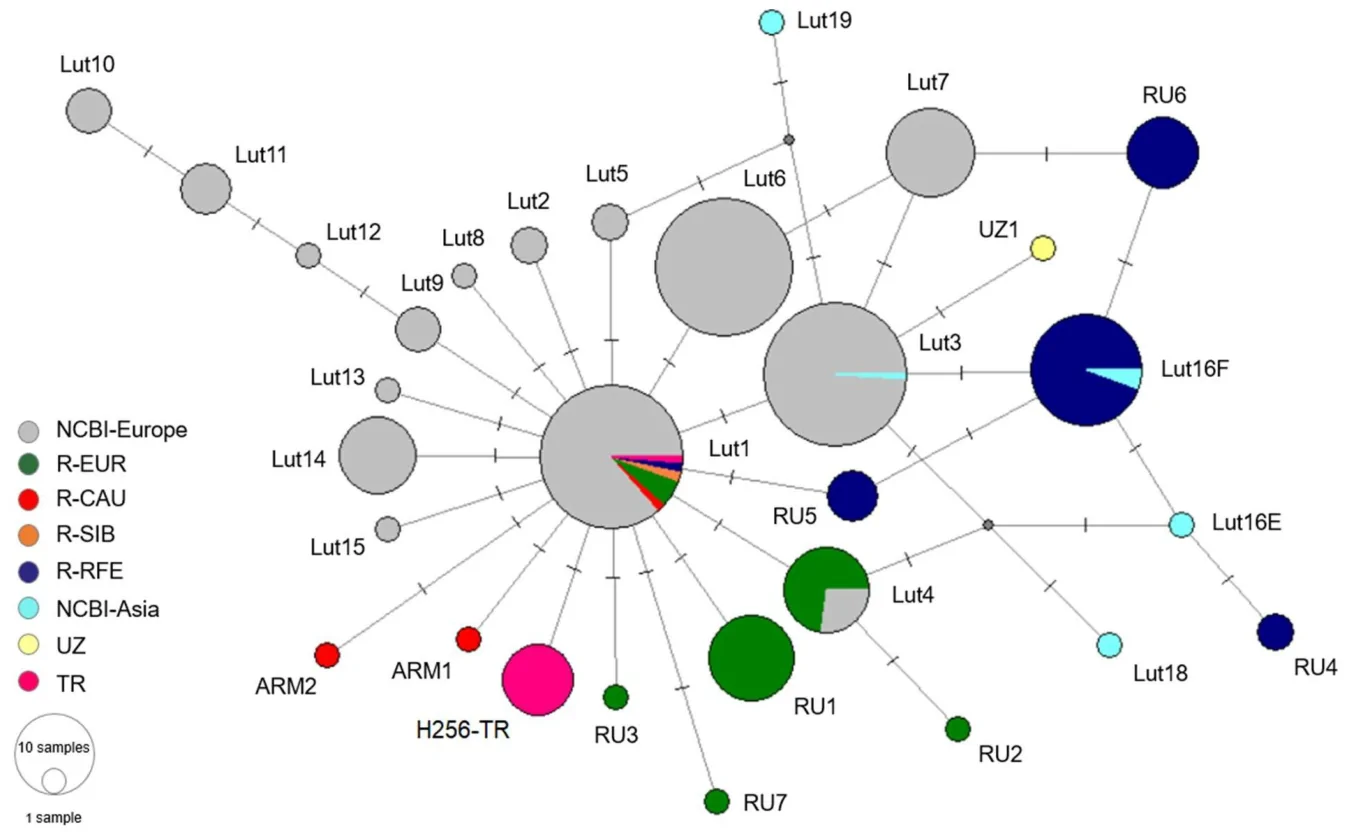
Median-joining network of the 256 bp fragment of the mtDNA control region of Eurasian otters (*L. lutra*). NCBI-Europe: European haplotypes from NCBI; R-EUR: European Russia; R-CAU: Caucasus (*L. l. meridionalis*); R-SIB: Siberia; R-RFE: Russian Far East; NCBI-Asia: otter haplotypes from South Korea, China and Japan from NCBI; and UZ: Uzbekistan (*L. l. seistanica*). The regional and haplotype-group abbreviations used in the network follow the nomenclature described in [44]. H256-TR denotes the novel haplotype identified in Türkiye. Circles indicate haplotypes and their relative frequencies in the different regions. Circle sizes are proportional to sample sizes, and only one substitution occurred between each pair of haplotypes.

**Figure 3:**
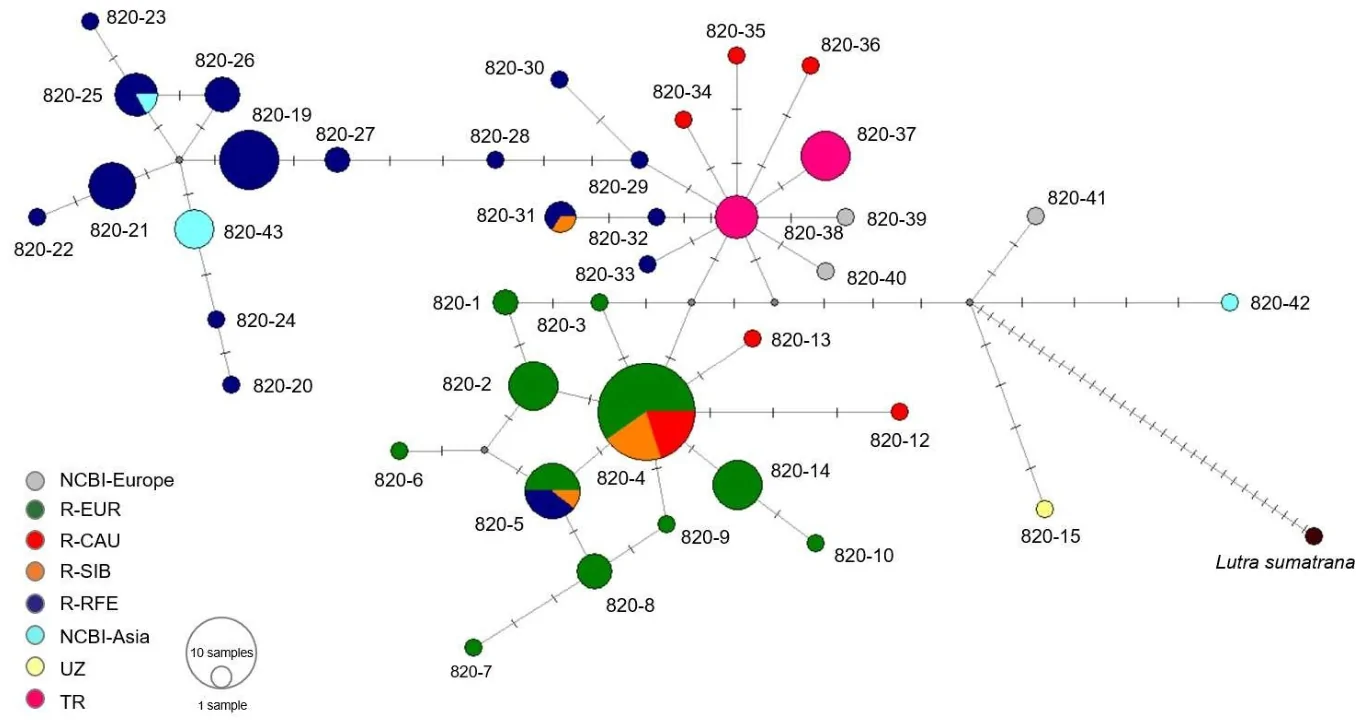
Median-joining network of 820 bp fragment of partial mtDNA of Eurasian otter (*L. lutra*). NCBI-Europe: European haplotypes from NCBI; R-EUR: European Russia; R-CAU: Caucasus (*L. l. meridionalis*); R-SIB: Siberia; R-RFE: Russian Far East; NCBI-Asia: Asian haplotypes from NCBI; and UZ: Uzbekistan (*L. l. seistanica*). The regional and haplotype-group abbreviations used in the network follow the nomenclature described in [44]. Circles indicate haplotypes and their relative frequencies in the different regions. Circle sizes are proportional to sample sizes. The number of mutation positions is indicated as hatch marks on the branches.

The two datasets with different mtDNA lengths formed two MJ networks of haplotypes with different topologies. The MJ network based on the 256-bp haplotypes did not reveal a clear phylogenetic structure (Figure 2). In contrast, the MJ network of the 820-bp sequences identified two weakly differentiated haplogroups, one comprising sequences from East Asia (Far Eastern Russia and Korea) and one comprising sequences from Northern Eurasia (Figure 3). The northern Eurasian phylogroup comprised primarily Anatolian, Caucasian, European Russian, and Northern European specimens in a close evolutionary relationship, with one or only a few substitutions among haplotypes. Both the East Asian and northern Eurasian haplogroups showed similarly shallow evolutionary intragroup divergence with a maximum of seven and nine substitutions, respectively, relative to the Far Eastern Russian haplotype H820-28 that connected the two groups. Notably, several Far Eastern Russian haplotypes (H820-29 to -33) were phylogenetically closely related to the European Russian haplotypes via the Central Anatolian haplotype H820-38, which formed the basis of a young star-like radiation leading to European haplotypes and other Russian haplotypes. The haplotypes of the Uzbek subspecies, *Lutra lutra seistanica*, and a European and an Eastern Asian specimen did not belong to any of these two shallow groups of haplotypes and appeared to be ancestral to both groupings, as inferred from the connecting position of the outgroup haplotype of *Lutra sumatrana*. The phylogenetic orientation obtained from our outgroup rooting was confirmed by a second MJ network constructed with one additional outgroup haplotype of *Enhydra lutris* (access. nr. AB291077).

**Figure 4:**
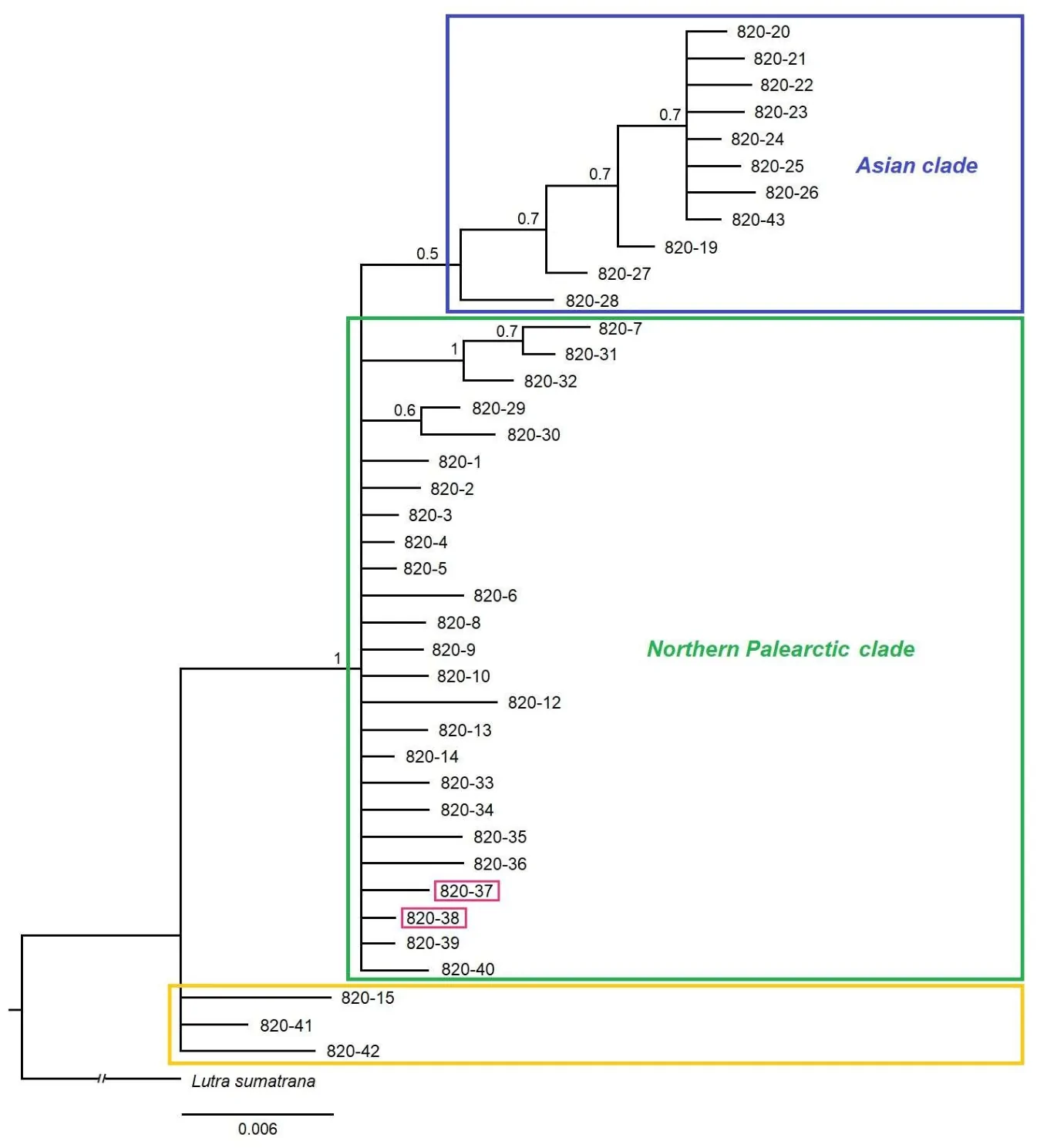
Bayesian Inference (BI) tree of the mt sequences (820 bp) based on the K81uf+I+G substitution model. *Lutra sumatrana* (GenBank accession number: NC_035810.1) was used as an outgroup. The blue box shows the Asian clade, the green box shows the Northern Palearctic clade, and the yellow box shows Uzbek, European, and Eastern Asian specimens that do not belong to any clade due to numerous mutational differences. The pink-framed haplotypes are novel Turkish haplotypes. Numbers at the nodes indicate Bayesian posterior probabilities (PPs).

The Bayesian phylogenetic tree constructed using the 820 bp sequences revealed a shallow topology with lower phylogeographic resolution than the MJ network. The only exceptions were three ancestral divergent haplotypes from China, Uzbekistan (*L. l. seistanica*), and Britain, i.e., the same haplotypes that also showed ancestral positions in the rooted MJ network of the long sequences. Apart from these three haplotypes, two major haplotype groups were weakly differentiated: a Far Eastern clade encompassing samples from Korea and the Russian Far East, and a Northern Palearctic clade containing haplotypes from other regions of Russia, Europe, Anatolia, and the Caucasus. High genetic similarity was observed among sequences from the Northwest Palearctic (Europe, Anatolia, the Caucasus, and European Russia). However, two small subgroupings emerged within the Northern Palearctic clade, likely due to the inclusion of individual samples from the Russian Far East. Furthermore, the Far Eastern clade received poor statistical support as a distinct monophyletic group (Bayesian posterior probability = 0.50). The Anatolian haplotypes identified in this study exhibited a close phylogenetic relationship with British and Caucasian haplotypes (Figure 3), but this could not be immediately inferred from the Bayesian tree (Figure 4).

## 4. Discussion

Our study represents the first non-invasive phylogeographic assessment of Eurasian otters (*Lutra lutra*) in Central Anatolia, revealing previously undocumented mitochondrial (mt) lineages in this key biogeographic region where Euro-Siberian, Mediterranean, Sindo-Turanian, and Afro-Arabian mammalian elements converge—e.g., [8,35,38,49,50]. Owing to its geographic position at the junction of southwestern Asia and Southeastern Europe, Anatolia has been considered a potential contact zone between European and Caucasian otter lineages [33]. Our findings support this view, suggesting Anatolia’s role as a phylogeographic bridge between lineages. By sequencing two mtDNA fragments from spraints collected along the Central Anatolian section of the Kızılırmak River, we identified three novel haplotypes, one (H256-TR) for the short (256 bp) sequence stretch and two (H820-37, H820-38) for the long sequence stretch (820 bp), in addition to the widespread Eurasian haplotype l for the 256 bp stretch. Our findings somewhat expand the hitherto sparse information on genetic variation of otters in Türkiye, and they are in line with the expectation that Anatolia may represent a reservoir of mt diversity. Our findings are also not incongruent with the hypothesis that Anatolia could have harbored a source population of otters for postglacial recolonization of Europe, basically along two main migration routes: one from northwestern Anatolia to the southeastern Balkans and another one from northeastern Anatolia along the late Pleistocene/early Holocene eastern seaboard of the Black Sea northward to the areas of today’s Georgia, southwestern and western Russia, Ukraine, and eventually other parts of Eastern Europe [33].

Moreover, our current sequence data, combined with all available mt alignments in GenBank, add to earlier taxonomic questioning of otters in the Caucasus region and other parts of the Middle East regarding their subspecies status of *L. l. meridionalis*. Our finding of new haplotypes, despite a relatively restricted sample size, from a catchment region in northern Central Anatolia may suggest relatively high genetic diversity in the mt genome of otters at that biogeographic crossroads. However, it also calls for a wider genetic survey of otters in Anatolia for a more comprehensive picture and for detailed implications for conservation of otters in the Near East. Our data establish a starting point for understanding otter phylogeography in southwestern Asia and Southeastern Europe and may trigger further studies of regional population structure of otters and associated conservation planning in Türkiye and other parts of the Near East.

### 4.1. Mitochondrial Haplotypes in Central Anatolia and Their Phylogenetic Link Between Asian and European Lineages

To date, only two distinct haplotypes have been identified for the complete cytochrome b (1140 bp) gene in three otters from the Kars, Sinop, and Istanbul regions of Türkiye [33]. One haplotype was found in the Anatolian samples, whereas the other was unique to the specimen from Turkish Thrace, i.e., the European part of Türkiye. For these Turkish samples, haplotype diversity (Hd) was calculated as 0.6667, and nucleotide diversity π as 0.00117. By supplementing these data with seven complete cytochrome b sequences in GenBank, a total of seven haplotypes were identified [33]. Notably, the Anatolian samples (from Sinop and Kars) shared the LLUTR1 haplotype with a GenBank sequence from Wetlina in southeastern Poland (Accession No. AB564050). Conversely, the haplotype of the sample from the European part of Istanbul was novel, having not been previously documented among the GenBank sequences. A phylogenetic analysis of these seven haplotypes with both diverse tree-building and median-joining (MJ) network analyses consistently revealed two primary haplogroups. One haplogroup comprised haplotypes from Türkiye, Iberia, Norway, and Poland, demonstrating high genetic affinity, while the other was formed exclusively by Korean haplotypes, which were distinctly separated from all other samples, indicating a divergent, unique lineage for Korean otters. The haplotype from Turkish Thrace (Istanbul) clustered more closely with Iberian samples, reflecting a phylogenetic connection between these European populations and the otter from Turkish Thrace. As proposed by [33], the genetic divergence observed among the three Turkish samples may reflect the presence of at least two distinct lineages in Türkiye. The study further suggested a post-glacial colonization scenario for Europe involving two major migration routes: a western route originating in the Balkans and northwestern Anatolia and ultimately reaching the Iberian Peninsula, and an eastern route originating in northeastern Anatolia and extending into Eastern Europe along the eastern Black Sea coast during the late Pleistocene and early Holocene, as well as through nearby inland migration corridors. However, to validate this phylogeographical hypothesis, sequence data from various mtDNA markers and nuclear DNA of individuals from a wider geographical range were recommended [33].

The MJ networks of both our presently found haplotypes for the 820 bp long mtDNA sequences and the short (256 bp) sequences are not incongruent with the hypothesis of such an Anatolian Late Pleistocene/early Holocene source population for the recolonization of Europe [33]. The presence of the widely distributed short-sequence haplotype Lut1 (our Lut1) in the Central Anatolian Kızılırmak catchment provides a phylogeographical link between two Caucasus lineages (ARM1, ARM2) and all corresponding documented European haplotype alignments. The longer fragment revealed two weakly separated phylogroups: an East Asian group (Korea, Russian Far East) and a Northern Palearctic group comprising Europe, European Russia, the Caucasus, and Anatolia. The Central Anatolian haplotypes clustered firmly within the Northern Palearctic group and showed particularly close affinity to the Caucasian and European sequences. Specifically, haplotype H820-38 connected, on the one hand, the Caucasian haplotypes and, on the other hand, the European, Caucasian, and Far Eastern haplotypes. This MJ network pattern may suggest the presence of a broader western Eurasian lineage system in Anatolia. The intermediate position of Anatolian haplotypes between Far Eastern and European as well as Caucasian lineages is consistent with the region’s role as biogeographic crossroads linking the Caucasus, the Balkans, and Southwest Asia. However, as expected by theory [40], this pattern was only apparent in the MJ network, whereas all our tree-building phylogenetic analyses indicated only two poorly supported (posterior probability, PP = 0.5) groupings, one Asian and one Northern Palearctic clade, but did not show any detailed phylogeographic resolution for the Northern Palearctic haplotypes.

### 4.2. Implications for Potential Postglacial Colonization Routes into Europe

Our findings do not contradict the hypothesis that Anatolia contributed to two postglacial migration routes into Europe, as previously proposed [33]. Specifically, the close phylogenetic relationship between haplotype H820-38 and two European haplotypes may suggest a western dispersal route from Anatolia or the late Pleistocene land-bridge region of the Bosphorous/Marmara Lake region via the (southern) Balkans. At the same time, the close phylogenetic affinity of Anatolian haplotypes to Caucasian lineages aligns with an eastern migration route through (the western foothills of) the Caucasus and northward along the eastern Black Sea coast or in hinterland waters. The presence of otters in the Terek and Kuban basins north of the Caucasus ([44], Figure 1; [51], Figure 2—contrary to many other range maps, as in, e.g., [39,52,53,54]) further supports the plausibility of this eastern migration corridor, particularly as they connect to other otter ranges further north in southwestern and western Russia and other parts of Eastern Europe.

Overall, both our tree-generating analyses and our MJ network for the longer sequences accord with a phylogeographic pattern as described by category V, a combination of categories III and IV, i.e., a shallow gene tree of closely related haplotypes, with geographic sympatric and allopatric distribution with no major phylogenetic gaps. This pattern describes closely related haplotypes that may be restricted to distinct geographic areas (e.g., as the currently observed Central Anatolian haplotypes H820-37 and -38) or may show widespread distribution and some geographic overlap [55]; e.g., the phylogenetically closely related haplotype H820-4 occurs in both Siberian and European Russia as well as the Caucasus but has not been detected among our Central Anatolian samples. For the question of the postglacial colonization of Europe, our short-sequence MJ network indicates the presence of Lut1 in western Russia, the Caucasus region, and Anatolia. This corresponds to scenarios of gene flow from Northwest Anatolia across the Bosphorus Sea of Marmara–Dardanelles waterway to Central and Western Europe via the southeastern Balkans. It does not contradict the hypothesis of gene flow from Northeast Anatolia to Eastern Europe via the Caucasus region and northward east of the Black Sea. However, it equally aligns with a converse model of postglacial gene flow from western Siberia to Europe and Anatolia via the Caucasus region.

Our MJ network based on the longer sequences is broadly consistent with those reported by [39] but differs somewhat from the topology described by [44]. It shows that the Central Anatolian haplotype H820-38 is the basis of a star-like radiation; the fact that this haplotype serves as a phylogenetic basis for two European haplotypes (H820-39, H820-40) and, independently, for three Caucasian haplotypes (H820-34, H820-35, H820-36) is in line with the hypothesis that Anatolia may have contributed to two postglacial migration routes into Europe, as previously proposed. The British haplotype H820-41 appears in a position ancestral to the Anatolian, Caucasian, and European haplotypes, together with an Uzbekistan haplotype (H820-15) and a Siberian haplotype (H820-42). Under the assumption of no historic anthropogenic translocations, the British haplotype H820-41 could indicate the presence of an ancestral Eurasian otter lineage in Europe derived from an eastern (western Asiatic?) source region. However, such translocations cannot be fully excluded ([39] and personal experience by one of the authors, FS, with an earlier forensic genetic case).

Apart from the haplotype H820-41, the Russian, western Siberian, and Caucasus haplotypes do not connect directly to European haplotypes. Notably, the Anatolian haplotype H820-38 also gives rise to all European Russian haplotypes and to all Caucasian haplotypes. This may suggest the role of Anatolia as a source region for postglacial colonization of Europe via the western Anatolian–southeastern Balkan route and possibly also via the route along the eastern Black Sea and the basins north of the Caucasus. A more comprehensive phylogeographic reconstruction, however, will ideally require both longer mt fragments and expanded sampling beyond Anatolia, including adjacent regions of the Middle East but also from the Caucasus and north of it—east of the Black Sea—specifically given the very few samples of European haplotypes (and samples) for that MJ model. Southeastern Balkan samples could particularly help elucidate whether late Pleistocene otters may have formed a long-term continuous population in the region of Turkish Thrace and Northwest Anatolia, whereas samples from the Terek and Kuban basins, north of the Caucasus, and further north in southwestern Russia and Ukraine would help reveal the extent of postglacial gene flow into Eastern Europe along the hypothesized eastern migration route.

The current otter distribution in northern Anatolia spans the northwestern and northeastern coastal areas, extending along the Black Sea coast of Turkish Thrace [9]. Prehistoric otter finds (from the Mesolithic onwards) from Romania [56] and Anatolia (see, e.g., [57] for the Neolithic village site of Erbaba Höyük and [58] for the middle Holocene occupation at the Alaybeyi Höyük in northwestern Anatolia) may suggest a wide late Pleistocene/early Holocene otter distribution across the southeastern Balkans and Anatolia. A late-glacial or postglacial expansion from such a potentially widespread refugial population is conceivable along the northwestern Anatolian Black Sea coast and across the region of today’s Bosphorus, Sea of Marmara, and Dardanelles. During the late Pleistocene and early Holocene, i.e., during lower sea levels, this region was characterized by narrow river valleys—including the precursors to the modern Bosphorus and the Strait of Çanakkale—and a brackish lake in the Marmara basin [59]; this environment—along with the Black Sea coastal countryside—could have provided very acceptable habitats for otters (perhaps similar to the early Holocene and today’s Baltic Sea coast). Given the rapid contemporary expansion of otter populations in Europe following the implementation of conservation measures [60], along with their documented presence at high northern latitudes in Europe and Siberia—e.g., [61] (Figure 314, p. 1300)—a swift postglacial northward migration and colonization of central and north-central Europe from such a hypothesized southeastern Balkans/northwestern Anatolian population is plausible. To date, however, there is no conclusive timeline for the postglacial colonization of different regions in Europe due to the relatively scarce and somewhat ambiguous published records of prehistoric otter finds. Although [62] suggested that otters reached central and north-central Europe only during the Holocene, the presence of *L. lutra* in faunal remains from the northern lowlands of continental Europe dated to the Late Pleistocene-Early Holocene transition indicates an earlier occurrence [63,64,65] (Table 1, p. 447). Similarly, records of *L. lutra* from northwestern Russia date to the Early Mesolithic, shortly after the onset of glacial retreat, whereas the earliest records from the Baltic Sea region are from the Late Mesolithic [66] (Figure 2, p. 7). Furthermore, in contrast to the Eurasian beaver, *Castor fiber*, otter bones have not been listed among the mammals from the Mesolithic assemblages of Star Carr and Thatcham in Britain [67,68] but were reported from the late-glacial Langwith cave in Britain [69] (Table 3.1, p. 70) and postglacial riverbeds and caves in association with humans in other locations in Britain [70,71], and early Holocene otter finds are also known from Ireland [72]. Specifically, these late Pleistocene/early Holocene/Mesolithic finds from the British Isles suggest that otters recolonized northern Europe relatively quickly with the establishment of suitable (freshwater) habitats after the withdrawal of the glaciers.

A potential postglacial northeastern Anatolian expansion along the Black Sea coast and the associated tributaries should not have been problematic for otters: due to the significantly lower sea levels of the late-glacial Black Sea, northern Anatolian rivers like the Kızılırmak extended further north than they do today, onto the exposed shelf. The late-glacial/early Holocene Black Sea was an isolated freshwater and later a brackish lake, and its Anatolian coastline was characterized by interconnected estuaries, delta systems, shallow estuary lagoons, and swamps, rather than a strict marine barrier, with a high coastal dynamic related to the rising postglacial sea level—e.g., [73] and the literature cited therein. This unique landscape provided principally ideal, contiguous habitats for otters, facilitating unobstructed migration along the entire southern Black Sea coast and northward along the eastern coast and north of the Caucasus in the diverse late-glacial/early Holocene rivers.

Earlier mtDNA sequence data indicated very low diversity in Western Europe, possibly due to a historical (late glacial) bottleneck, e.g., [74,75,76,77,78,79], while diversity was higher in Belarus, western Russia, and further east in Asia—e.g., [68,80]. This corresponds in principle to either a single postglacial expansion scenario from Southeastern Europe/northwestern Anatolia or a scenario of northeastward expansion from Anatolia east of the Black Sea that could have merged with a western Russian gene pool, which might not have reached further west in Eastern Europe. Our currently presented hypothetical postglacial expansion scenario from Northwest Anatolia resembles the colonization history of Central, northern Central, and Western Europe by brown hares (*Lepus europaeus*), which expanded northward from late Pleistocene refugia in the southern and southeastern Balkans—a region that had received Anatolian lineages, which, however, remained restricted to the southeastern Balkans, e.g., [81].

### 4.3. Taxonomic Considerations, Conservation Implications, and Middle Eastern Genetic Landscape of Otters

Morphological analyses [32] and recent SNP-based studies [39] have questioned the validity of the subspecific status of *Lutra lutra meridionalis* from the Caucasus region (Georgia, Armenia, Azerbaijan) and Iran. Our mt data does not show any meaningful phylogenetic gap between the nominal subspecies *L. l. lutra* and *L. l. meridionalis*. In fact, the long-sequence haplotype H820-38 from Central Anatolia even connects Caucasian haplotypes, while it is closely connected to European haplotypes. This phylogenetic pattern supports the interpretation that Caucasian, Anatolian, and European otters belong to a continuous western Eurasian lineage system, i.e., lineage 3 of [39], with the inclusion of otters from Iraq in this lineage as well, rather than representing separate subspecies. However, definitive taxonomic conclusions will require nuclear genomic data and broader geographic sampling.

Today, the Kızılırmak River population faces multiple anthropogenic threats, including dams (see Figure 1), habitat fragmentation, pollution, and possibly also direct anthropogenic mortality caused by road casualties and poaching. The occurrence of unique haplotypes in this region highlights its role as a reservoir of genetic diversity and underscores the need to maintain habitat connectivity throughout the catchment. Protecting riparian habitats, especially by preserving complex riparian structure (e.g., [1,25,30]), maintaining migration corridors, and mitigating dam-related barriers would strengthen connectivity across the catchment, promote genetic exchange, and reduce the risk of declining effective population sizes. Given the species’ sensitivity to water quality and habitat degradation, the genetic distinctiveness documented presently should be incorporated into regional conservation planning along the river and its tributaries.

Our detection of both a Central European mtDNA CR haplotype and two novel haplotypes closely related to Caucasian lineages within the Kızılırmak River may suggest a genetically diverse population in Central Anatolia. Since the Kızılırmak River does not connect major eastern and western river catchments in Anatolia—such as the Sakarya River catchment west of it, the Euphrates basin in the east and the Aegean basins with the Gediz and Büyük Menderes rivers in the west—a more pronounced genetic differentiation across Anatolia may be expected. However, we can only speculate that the Black Sea coast may have provided connecting habitats between diverse late-glacial river courses via their deltas and estuaries, and inland waters close to the receding coastline with the postglacially rising sea level, perhaps like today’s southern Baltic Sea coast and its inland waterways—e.g., [82]. Given our limited sample size, broader sampling across Türkiye is necessary to map spatial patterns of gene pool diversity in Anatolian otters and to further test the phylogeographic hypothesis [33] mentioned above. Specifically, including samples from the Black Sea coast, the northern Aegean coast, the Marmara Sea, and northeastern Anatolia—analyzed in conjunction with reference sequences from the Balkans, southwestern Russia, and Ukraine—is essential to understanding whether and to what extent (late Pleistocene) Anatolian lineages may have proceeded northward in Europe. Ideally, nuclear markers (SNPs, microsatellites) should be included. Such a comprehensive approach will help resolve the late Pleistocene/early Holocene role of Anatolia in the maintenance and distribution of genetic diversity of an important flagship and umbrella species for freshwater habitats in the Middle East and its younger evolutionary relationship with northern and Western Palearctic populations.

## 5. Conclusions

Our non-invasive phylogeographic assessment of Eurasian otters in Central Anatolia reveals previously undocumented mitochondrial diversity in this key biogeographic region. By sequencing two mtDNA fragments from spraints collected along the Kızılırmak River, we identified one novel haplotype (H256-TR) for a short (256 bp) sequence stretch, in addition to the widespread Eurasian haplotype Lut1 and two novel haplotypes (H820-37, H820-38) for a long (820 bp) sequence fragment. These findings expand the hitherto little-known genetic variation of otters in Türkiye and may hint towards Anatolia functioning as a reservoir of mitochondrial genetic diversity. The topology of our short-sequence-based MJ network and of our tree-building dendrograms failed to resolve regional structure, likely due to insufficient mutational variation. Nevertheless, the short-sequence-based network indicated the close evolutionary relationship of the Anatolian haplotypes with two of three European haplotypes. In fact, one central haplotype of European otters was also shared by Anatolian and Caucasian (Armenian) otters. In contrast, the MJ network for the longer fragment showed somewhat better-defined haplogroups and revealed phylogeographically meaningful relationships among Anatolian, Caucasian, and European haplotypes, even though they also showed only shallow global evolutionary divergence. Our tree-building models for the longer fragments produced only two marginally supported clades, an Asian clade (Korea, Russian Far East) and a Northern Palearctic clade. The latter included all haplotypes from Europe, the Caucasus, and Anatolia, in addition to Russian haplotypes. The presently revealed phylogeographic pattern supports the view that Anatolia forms part of a broader western Eurasian lineage system rather than harboring a divergent eastern lineage. It also indicates the intermediate position of Anatolian haplotypes between Far Eastern and European lineages, consistent with the region’s role as a biogeographic crossroads linking the Caucasus, the Balkans, and Southwest Asia. The presence of both the widespread Lut1 haplotype and some novel haplotypes within a relatively small geographic area indicates that the Kızılırmak basin may represent a zone of historical admixture or a late Pleistocene refugium during the Last Glacial Maximum. Moreover, one Anatolian haplotype was found among the currently studied haplotypes to represent the only basis of two long-fragment haplotypes of European otters; the same Anatolian haplotype connects phylogenetically Caucasian and European haplotypes. This is not incongruent with the earlier hypothesis that Anatolia may have functioned as a source population of otters expanding northward to Europe (see also [83]), potentially via the Balkan route and by an eastern route along the eastern Black Sea coast and adjacent waterways more inland [33]. Our phylogeographic findings are also in line with the earlier view that, based on morphometric [32] and SNP [39] data, the subspecies status of *Lutra l. meridionalis* is questionable. Given that the Kızılırmak River has no connection to other Anatolian water catchments, a wider geographical sampling across Anatolia would help provide a more comprehensive phylogeographic picture of otters in the Middle East and may allow further testing of the hypothesis of postglacial northward expansion and thereby contribute to our understanding of the postglacial colonization of Europe by otters.

## Figures and Tables

**Table 1:** Localities where otter spraints were collected in the study area.

Localities	Coordinates
A. Kırıkkale/Sulakyurt-Hamzalı Village	40°18′52.54″ N–33°39′26.49″ E
B. Ankara/Kalecik (Güriş Hydroelectric Power Plant)	40°03′46.48″ N–33°26′44.86″ E
C. Kırıkkale/Hacılar (Kapulukaya Dam)	39°43′46.44″ N–33°28′53.27″ E
D. Ankara/Bala (Kesikköprü Dam)	39°23′49.91″ N–33°25′17.86″ E
E. Kırşehir/Kaman (Hirfanlı Dam)	39°16′26.36″ N–33^°^31′05.94″ E

**Table 2:** Haplotypes of two mtDNA fragments (256 bp vs. 820 bp) based on locality and the number of spraints in which they were determined. The numbers in parentheses indicate the number of spraint samples in which the haplotype was determined. The “+” sign indicates the presence of the haplotype at the relevant locality, while the “-” sign indicates its absence.

The Length of DNA Fragments and Haplotypes				
		256 bp	820 bp	
Localities	H256-TR	Lut1	H37	H38
A. Kırıkkale/Sulakyurt	+ (1)	-	+ (1)	-
B. Ankara/Kalecik	+ (1)	+ (1)	+ (1)	+ (1)
C. Kırıkkale/Hacılar (Kapulukaya Dam)	-	+ (2)	-	+ (2)
D. Ankara/Bala (Kesikköprü Dam)	+ (5)	+ (3)	+ (5)	+ (3)
E. Kırşehir/Kaman (Hirfanlı Dam)	+ (1)	-	+ (1)	-

## Data Availability

The mtDNA sequences generated in this study have been deposited in GenBank under the accession numbers provided in the Supplementary Materials.

## Supplementary Materials

The following supporting information can be downloaded at https://www.mdpi.com/article/10.3390/ani16213371/s1: Table S1 NCBI GenBank accession numbers and relevant references for the sequences used in the short-sequence (256 bp) analyses; Table S2: NCBI GenBank accession numbers and relevant references for the sequences used in the long-sequence (820 bp) analyses (The reference list is presented in Table S1).
